# Roles of mycorrhizal fungi on seed germination of two Chinese medicinal orchids: need or do not need a fungus?

**DOI:** 10.3389/fpls.2024.1415401

**Published:** 2024-07-30

**Authors:** Jing Yang, Neng-Qi Li, Jiang-Yun Gao

**Affiliations:** Institute of Biodiversity, School of Ecology and Environmental Science, Yunnan University, Kunming, Yunnan, China

**Keywords:** traditional medicinal plants, mycorrhizal fungi, orchid conservation, symbiotic seed germination, *Serendipita*, *Pleione bulbocodioides*, *Bletilla striata*

## Abstract

Generally, orchids highly depend on specific fungi for seed germination and subsequent seedling development in nature. For medicinal orchids, obtaining compatible fungi is prerequisite for imitation of wild cultivation and conservation. In this study, the two important traditional Chinese medicinal orchids, *Pleione bulbocodioides* and *Bletilla striata*, were studied to screen out effective fungi for seed germination and seedling development. *P. bulbocodioides* seeds germinated and formed protocorms in all fungal and control treatments, but seedlings only developed in fungal *Serendipita officinale* (SO) and *S. indica* (SI) treatments and nutrient-rich medium MS treatment. At 90 days after incubation, the percentages of seedlings were 34.83 ± 3.4% and 27.59 ± 3.5% in SO and SI treatments, which were significantly higher than the MS treatment (18.39 ± 2.0%; all *P* < 0.05). At this stage, most seedlings in SO and SI treatments bore two leaves (Stage 5), and pelotons inside the basal cells of seedlings were clearly observed. For *B. striata*, seeds germinated up to seedlings with or without fungus, but seedlings developed rapidly in SI treatment. At 90 days after incubation, the percentage of seedlings in SI treatment reached 77.90 ± 4.1%, but was significantly lower than the nutrient-poor medium OMA treatment (85.18 ± 3.7%; *P* < 0.01), however, the seedlings in SI treatment were stronger than the seedlings in OMA treatment. The results suggested that *P. bulbocodioides* rely on compatible fungi for seeds germinated up to seedlings, and fungus SO could effectively promote seed germination and support seedling development; while *B. striata* can germinate up to seedling without any fungus, but compatible fungus *S. indica* can greatly speed up seed germination and promote seedling development. We suggest that *S. officinale* and *S. indica* fungi can be used in conservation practices or imitation of wild cultivation of these two important medicinal orchids, respectively.

## Introduction

Orchids have high species diversity and widely distributed in almost all land-ecosystems (Cribb et al., 2003). In different cultures and countries, large numbers of orchid species have a long history of use as traditional medicines, health food supplements, or nutritional sources (see reviewed by Hossain, 2011), and these orchids have been massively collected (Ghorbani et al., 2014; Liu et al., 2014). Globally, over-exploitation is one of the major impacts driving many orchid species to become rare and endangered, and some species even have been extinct from the wild (Swarts and Dixon, 2009; Fay, 2018; Wraith and Pickering, 2019). Therefore, orchids are among the most threatened of all flowering plants and have been considered as flagship species for plant conservation globally (Baillie et al., 2004; Fay, 2018).

China is one of the orchid-rich countries with 1708 species, has a very long history in using many orchid species as traditional Chinese medicines (TCM) (Zhou et al., 2021). About 350 orchid species are used in TCM, 97 of which are Chinese endemics (Liu et al., 2014). Beside the well-known *Dendrobium* species (Shi-Hu in Chinese) and *Gastrodia* species (Tian-Ma in Chinese), other orchids, such as species of *Pleione* and *Bletilla* are also widely used in TCM (The State Pharmacopoeia Commission of P. R. China, 2010). Without exception, such medicinal orchids have been over-collected for a long time, and many species even reached to the point of local extirpation (Liu et al., 2014). Fortunately, in the newly issued National Key Protected Wild Plants, about 350 orchid species from 23 genera have been listed, which includes some over-collected medicinal and/or ornamental genera such as *Anoectochilus*, *Cymbidium*, *Cypripedium*, *Dendrobium*, *Paphiopedilum*, *Pleione* and *Renanthera*. All species of the genera are listed as class I and II of protection categories (http://www.forestry.gov.cn/main/5461/20210908/162515850572900.html).

For those endangered medicinal plants, the concept of restoration-friendly cultivation or imitation of wild cultivation, in which medicinal plants are cultured in natural forests, has been proposed to achieve effective conservation and sustainable utilization (Huang and Guo, 2009; Liu et al., 2014). The idea is increasingly recognized by practitioners, and developing different cultivation modes is also encouraged by China government. The imitation of wild cultivation of Chinese medicinal materials has now become an important direction for the sustainable and healthy development of Chinese herbal medicine industry, and has also been considered as an important way in the future to link the commercial TCM industry together with initiatives of biodiversity conservation in China (Cheng et al., 2019).

For medicinal orchids, it is quite difficult to develop a successful mode for such imitation of wild cultivation in natural condition, because most of orchid species highly depend on specific fungi for seed germination and subsequent seedling development (Arditti and Ghani, 2000; Rasmussen et al., 2015). Although, asymbiotic seed germination is still the most straightforward way of producing seedlings in large quantities and has been widely used in many orchids for commercial seedling production (Chen et al., 2015), symbiotic seed germination has practical merits for species conservation (e.g., Stewart et al., 2003; Batty et al., 2008; Otero et al., 2013). It could also be particularly important for imitation of wild cultivation for orchids, as in medicinal *Dendrobium* species and other endangered orchids, germinating seeds together with compatible mycorrhizal fungi resulted in a success of obtaining massive seedlings with low-cost and seedlings better adapting to the environment with higher survivorship and fast growth (Wang et al., 2021; Yang et al., 2023). Obtaining compatible fungi and using fungi to facilitate seed germination in practice are two key steps for imitation of wild cultivation or reintroduction based on symbiotic seed germination (Zhou and Gao, 2011; Yang et al., 2023).

*Pleione bulbocodioides* (Franchet) Rolfe is a terrestrial or lithophytic orchid widely distributed in subtropical area or temperate zone in China (Chen et al., 2009). It is a traditional Chinese medicinal orchid, and its tuber is commonly known as Bing-Qiu-Zi in folk. Its tuber is used to clear and detoxify heat and eliminate swelling and pain, and is also the main ingredient of several Chinese patent drugs (The State Pharmacopoeia Commission of P. R. China, 2010). *Bletilla striata* (Thunberg) H. G. Reichenbach is a terrestrial orchid widely distributed in subtropical area with altitude of 100-3200 m in China (Chen et al., 2009), and is also a traditional Chinese medicinal orchid, and its tuber is commonly known as Bai-Ji which has been used to treat tuberculosis, hemoptysis, gastric, and duodenal ulcers (He et al., 2017). In the local medicine markets, the dried tubers of *Bletilla striata* are often used to sell as fake Bing-Qiu-Zi (Zhou, 2001). Because it already formed a large Bai-Ji industry in China, the market price of Bai-Ji is much cheaper than Bing-Qiu-Zi.

There are relatively few studies on mycorrhizal associations in *Pleione* species. Based on ITS-rDNA sequencing for mycobionts of 15 *Pleione* species, Tulasnellaceae, Ceratobasidiaceae, Serendipitaceae (Sebacinales), Atractiellales, and Auriculariales were reported as putative mycobionts of *Pleione*, and different *Pleione* species with a sympatric distribution showed preferences for different fungi (Qin et al., 2019). By continuous samplings during a whole root lifecycle, a interesting study released that plants of *P. bulbocodioides* could be quickly colonized by OMF at root emergence and had a constant OMF composition throughout one root lifecycle, although the OMF richness declined with root aging after a peak occurrence during root elongation, the richness of root-inhabiting fungal endophytes kept increasing with root aging and more drastic turnovers were found in their species compositions (Qin et al., 2021). In an early study, 9 non-OMFs belonging to *Trichoderma*, *Paecilomyces* and *Fusarium* were isolated from roots of *P. bulbocodioides*, and found that all 9 fungal strains could stimulate seeds germinated to protocorms (Yang et al., 2008).

As a very popular medicinal orchid, *B. striata* has received much research attentions including symbiotic seed germination. Interestingly, many studies found that a wide range of fungi including orchid mycorrhizal fungi (OMF) and non-OMFs, could promote seed germination and seedling development in *B. striata* (e.g., *Fusarium oxysporum*, Jiang et al., 2019; *Coprinus* sp., *Tulasnella* sp., *Sebacina* sp. and *Serendipita* sp., Xu et al., 2019; *Serendipita* sp. and *Schizothecium fimbriatum*, Xi et al., 2020; *Tulasnella* spp., Yamamoto et al., 2017; Fuji et al., 2020; Liu et al., 2022). It seems that any fungi can promote seed germination and seedling development in *B. striata.* However, an early study revealed that, unlike most other orchids, seeds of *B. striata* contain a large amount of starches and lipids which can provide nutrients for seed germination (Guo and Xu, 1990). The same researchers also suggested that fungi (*Mycena osmundicola*, etc) could promote cotyledon differentiation and growth, as well as rhizoid formation of *B. striata* protocorms after seeds are sown with fungi (Guo and Xu, 1992). From these results, it is still unclear if seeds of *B. striata* can autonomously germinate up to seedlings, and can fungus *Serendipita indica* aid to seed germination and subsequently seedling development in *B. striata*? Because *S. indica* has been tested to be compatible to many orchid species and could promote their seed germination and seedling development (Xu et al., 2023). We are also curious if seeds of *P. bulbocodioides* can germinate up to seedlings without any fungi, or associate with different fungi to promote seed germination?

In this study, we compared the effects of four mycorrhizal fungi on seed germination and seedling development of *P. bulbocodioides*, and also comparably studied the seed germination and seedling development of *B. striata* by incubating seeds with and without fungus. The aims of this study are to screen out fungi which can effectively promote seed germination of *P. bulbocodioides* to aid further conservation and imitation of wild cultivation for this endangered medicinal orchid; and to confirm if seed germination of *B. striata* require any fungal association.

## Materials and methods

### Plant species and mycorrhizal fungi

**Figure 1:**
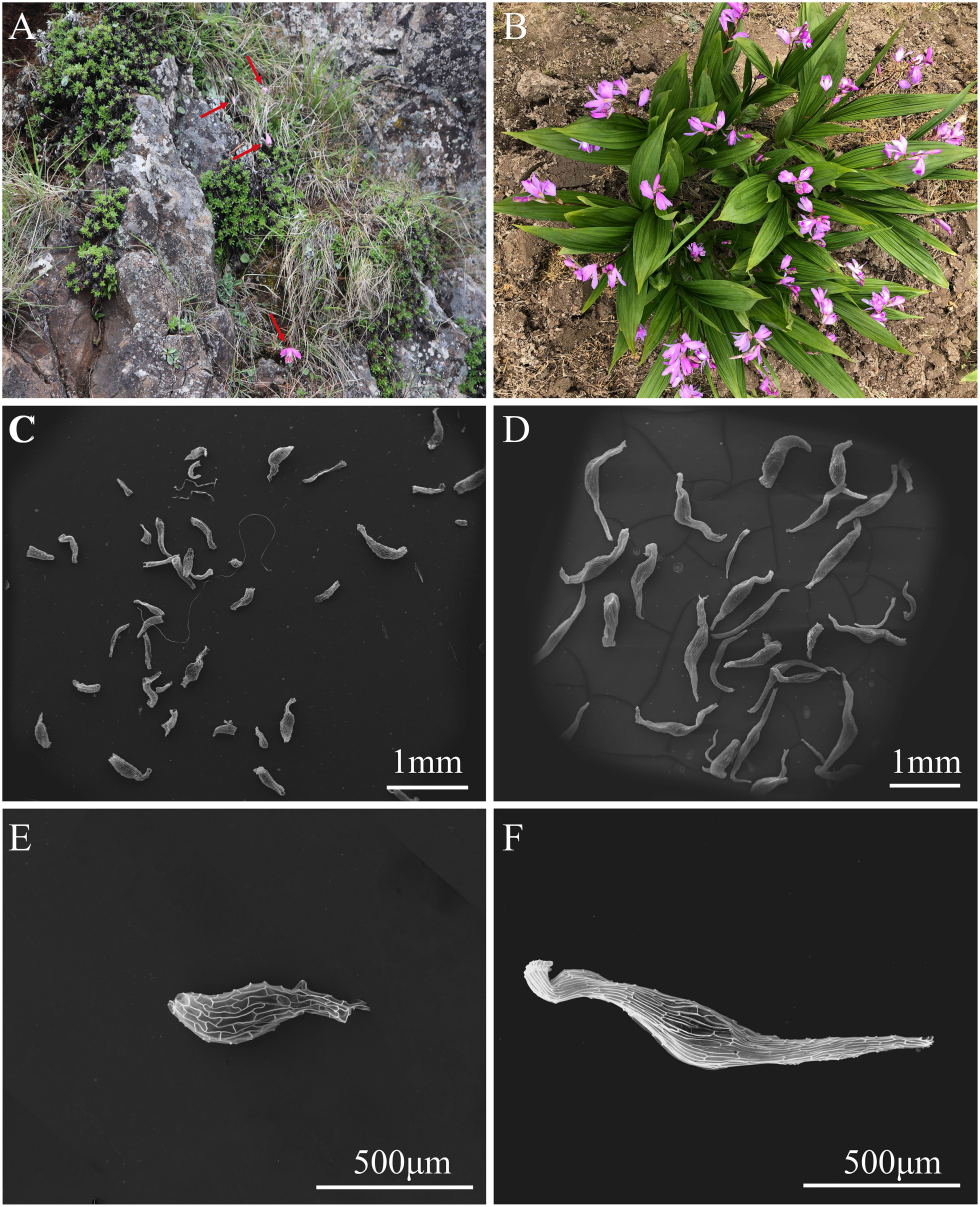
Plants and seeds of *Pleione bulbocodioides* and *Bletilla striata*. **(A)** Plants *of P. bulbocodioides* grows on mossy rocks with red arrows indicting flowers; **(B)** Cultivated plants of *B. striata*; **(C)** Seeds of *P. bulbocodioides*; **(D)** Seeds of *B. striata*; **(E)** A seed of *P. bulbocodioides*; **(F)** A seed of *B. striata*.

*Pleione bulbocodioides* usually grows in humus-covered soil, on mossy rocks (**Figure 1A**) with altitude from 900 to 3600 m (Chen et al., 2009). It flowers from April to June, and fruits get mature from October to November in Yunnan. Three naturally set fruits of *P. bulbocodioides* were harvested from wild plants in November 2022. It flowers during April and May, and fruits get mature during September and October. As an important TCM plant, *B. striata* now has been commonly cultivated (**Figure 1B**). About 30,000 mature fruits of *B. striata* resulted from artificial cross-pollination on cultivated plants were collected in Oct. 2021. For all collected fruits of the two orchids, seeds were carefully released and then dried and stored in the Orchid Seed Bank of Yunnan University following our previously established method (Gao et al., 2014). Prior to use, seeds were tested using the 2,3,5-triphenyl tetrazolium chloride (TTC) method to ensure high seed viability of over 90% (Vujanovic et al., 2000).

In this study, four mycorrhizal fungal strains were used to compare their effects on the seed germination and seedling development of *P. bulbocodioides*. The original sources and related information about the four fungal strains are summarized in **Table 1**. Among them, *Serendipita officinale* SO, *Tulasnella* sp. GYBQ01 and *Tulasnella* sp. Agp-1 were obtained from our previous studies. *Serendipita indica* SI (syn. *Piriformospora indica*; DSM11827) was originally obtained from the Leibniz Institute DSMZ-German Collection of Microorganisms and Cell Cultures, Braunschweig, Germany, and the identity has also been confirmed again by ITS sequence. *S. indica* has been considered as an endophytic fungus, and can associate with a variety of plant species to increase host plant tolerance to abiotic stresses (Wan et al., 2024).

**Table 1:** The four fungal strains used in this study with the information of original sources and GenBank accession number.

Fungal codes	Fungi species	Original sources	GenBank accession number	References
SO	*Serendipita officinale*	Protocorms and roots of *Dendrobium officinale*	MN173026	Wang et al., 2021; 2023
SI	*Serendipita indica*	Rhizosphere soil of desert shrubs	DSM11827	Verma et al., 1998
GYBQ01	*Tulasnella* sp.	Protocorms of *Paphiopedilum spicerianum*	MN733451	Yang et al., 2020
Agp-1	*Tulasnella* sp.	Protocorms of *Arundina graminifolia*	MK651837	Meng et al., 2019

### Seed morphology

Seed morphology of *P. bulbocodioides* and *B. striata* were compared using a scanning electron microscopy (SEM; FEI Quanta FEG 650, Thermo Fisher, American). The dried seeds were removed from refrigerator and stuck on the sample table, and then sputter coated with goldpalladium for 40s using a Quorum Q150R high vacuum sputter coater prior to SEM inspection. The seeds of the two species were photographed, and the number of cells on the longitudinal axis of each seed were counted. The length and width of the seed were measured using ImageJ, and compared using one-way ANOVA between two species.

### Effectiveness of fungal strains on seed germination and seedling development

Seeds of *P. bulbocodioides* and *B. striata* were sterilized with 2% (w/v) sodium hypochlorite solution (NaClO) for five minutes and then washed with sterile distilled water 3-5 times. Sterilized seeds were suspended in 0.1% agar solution and kept *ca*. 30 seeds for each 300 μL agar solution. A circular nylon cloth with a radius of 6 cm was placed on each oatmeal agar medium (OMA) of Petri dish, and 300 μL seed-agar suspensions were transferred onto the nylon cloth using a pipette.

For *P. bulbocodioides*, four fungal treatments and two control treatments were conducted. The four fungal treatments were fungal SO, GYBQ01, Agp-1 and SI treatments, in which one 0.5 cm^3^ piece of corresponding fungal inoculum was place in the center of the Petri dish for each of treatment. The two control treatments were conducted on two media without fungal strain, which were OMA treatment (nutrient-poor medium; Oatmeal agar medium) and MS treatment (nutrient-rich medium; Murashige and Skoog, 1962). For *B. striata*, two treatments, fungal SI treatment and OMA control treatment were conducted. All above treatments were replicated in 30 Petri dishes and incubated at 25 ± 2°C and 12/12-h light/dark cycle in germination chambers (RXZ300B, Ningbo Southeast Instruments Co., Ltd, Ningbo, China).

### Observations on symbiosis establishment

To determine if mycorrhizal symbiosis had been successfully established, the symbiotic protocorms or seedlings were randomly selected at different stages at 30, 60 and 90 days after incubation to examine the formation of pelotons. The samples were cleared using 10% KOH solution at 90°C for 30 min, and treated with 3% H_2_O_2_ solution for 3-4 min, washed with 1% HCl solution, and then stained in 0.05% (w/v) trypan blue in lactic acid glycerol solution at 37°C for 30 min (adapted from Phillips and Hayman, 1970), and then de-stained in acetic glycerol solution before observation under the microscope (DM2000, Leica Microsystems GmbH, Wetzlar, Germany). In addition, transverse sections of 4-μm thickness were cut from the LR white-embedded samples using an ultra-microtome (LEICA RM2245), and stained with 1% (w/v) toluidine blue, and then used to observe and photograph the formation of pelotons inside cells.

### Data collection and statistical analysis

Seed germination and seedling development are divided into five stages as: Stage 0, non-germination; Stage 1, embryo swells and turns green, and testa is propped up (germination); Stage 2, continued embryo enlargement forms a spherule, seed coat broken (protocorm formation); Stage 3, appearance of protomeristem (protocorm differentiation); Stage 4, seedling with emergence of first leaf; Stage 5, emergence of second leaf and further development (Arditti, 1967). For each Petri dish, the number of seeds and the status of seed germination were assessed and recorded under a dissection microscope at 30, 60 and 90 days after incubation. We used Stages 0, 1, (2 + 3) and (4 + 5) to determine no germination, seed germination, protocorm formation and seedling development, respectively. The number of total seeds (*t*), germinated seeds (*g*), protocorms (*p*), and seedlings (*s*) were used to calculate the percentages of germinated seeds (*G*), protocorms (*P*), and seedlings (*S*) as: *G* = (*g* + *p* + *s*)/*t*, *P* = (*p* + *s*)/*t*, and *S* = *s*/*t*, respectively.

All data are presented as mean ± standard error (SE). Effects of fungal inoculation on seed germination, protocorm formation and seedling development were compared using Generalized Linear Models (*GLM*s). The mean value of each treatment was tested pairwise using Least Significant Difference (*LSD*). In addition, One-way ANOVA was used to analyze the data of the two treatments for *B. striata*. All statistical analyses were performed in SPSS 25.0 (SPSS Inc., Chicago, USA) and graphs were made using SigmaPlot 13.0 (SYSTAT Inc., Chicago, USA).

## Results

### Seed morphology

Seeds of *P. bulbocodioides* were nearly white, and its shape was somewhere between spoon and spindle shaped (**Figure 1C**), while seeds of *B. striata* were light yellow, and spindle-shaped (**Figure 1D**). A seed of *P. bulbocodioides* contained 7-9 cells with 696.48 ± 85.0 µm in length and 166.25 ± 4.7 µm in width, which was significantly smaller than the seed of *B. striata* containing 9-11 cells with 1395.89 ± 74.0 µm in length and 205.00 ± 4.1 µm in width (n = 12, all *P* < 0.05; one-way ANOVA; **Figures 1E, F**).

### Effectiveness of fungal strains on seed germination and seedling development in *Pleione bulbocodioides*

**Figure 2:**
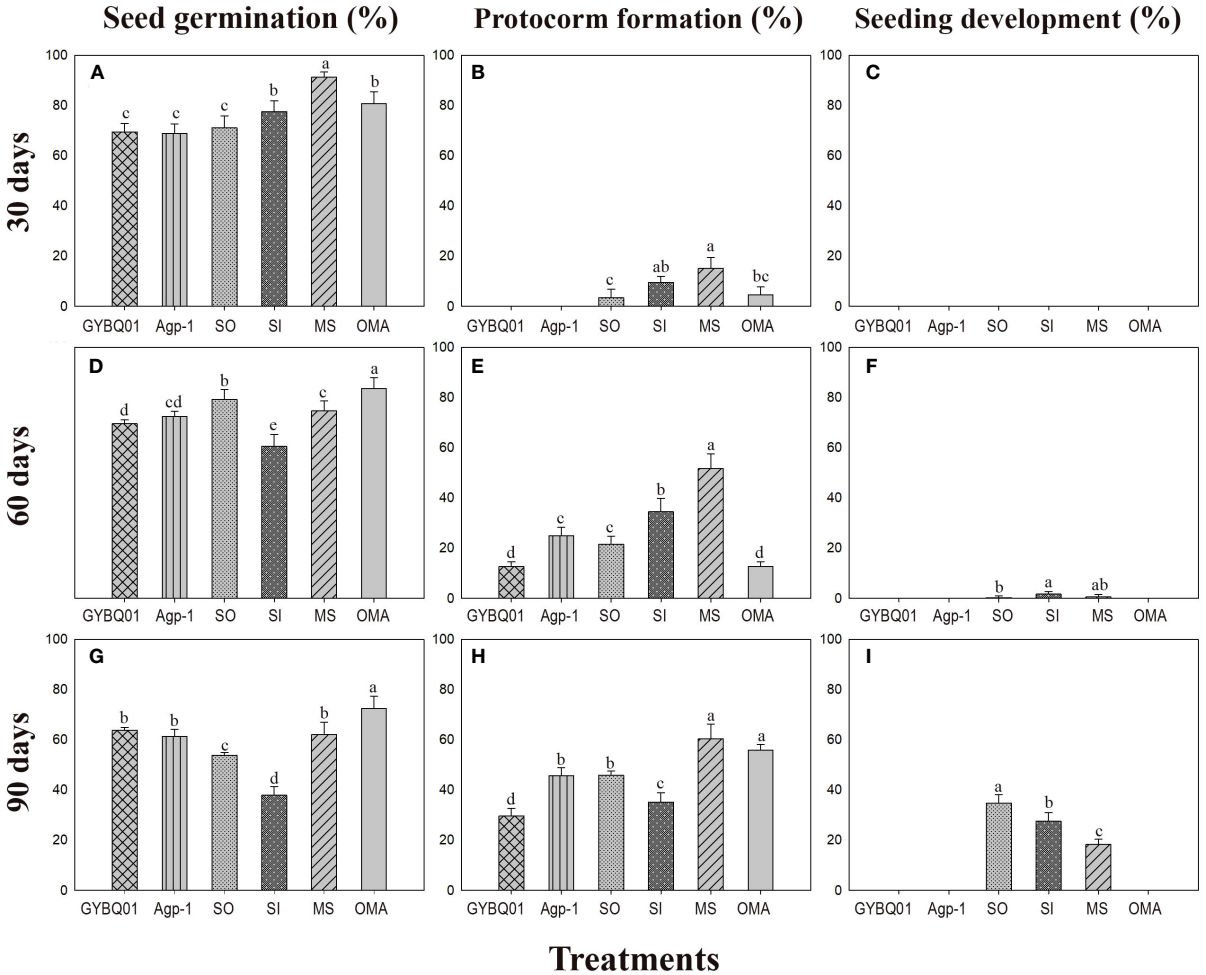
The percentages of seed germination, protocorm formation and seedling development (mean ± SE) in four fungal treatments (fungal SO, GYBQ01, Agp-1 and SI) and two control treatments (OMA and MS) of *Pleione bulbocodioides* at 30, 60 and 90 days after incubation. In each panel, different letters indicate significant differences (*p* < 0.05) based on the Generalized Linear Models (*GLM*s).

**Figure 3:**
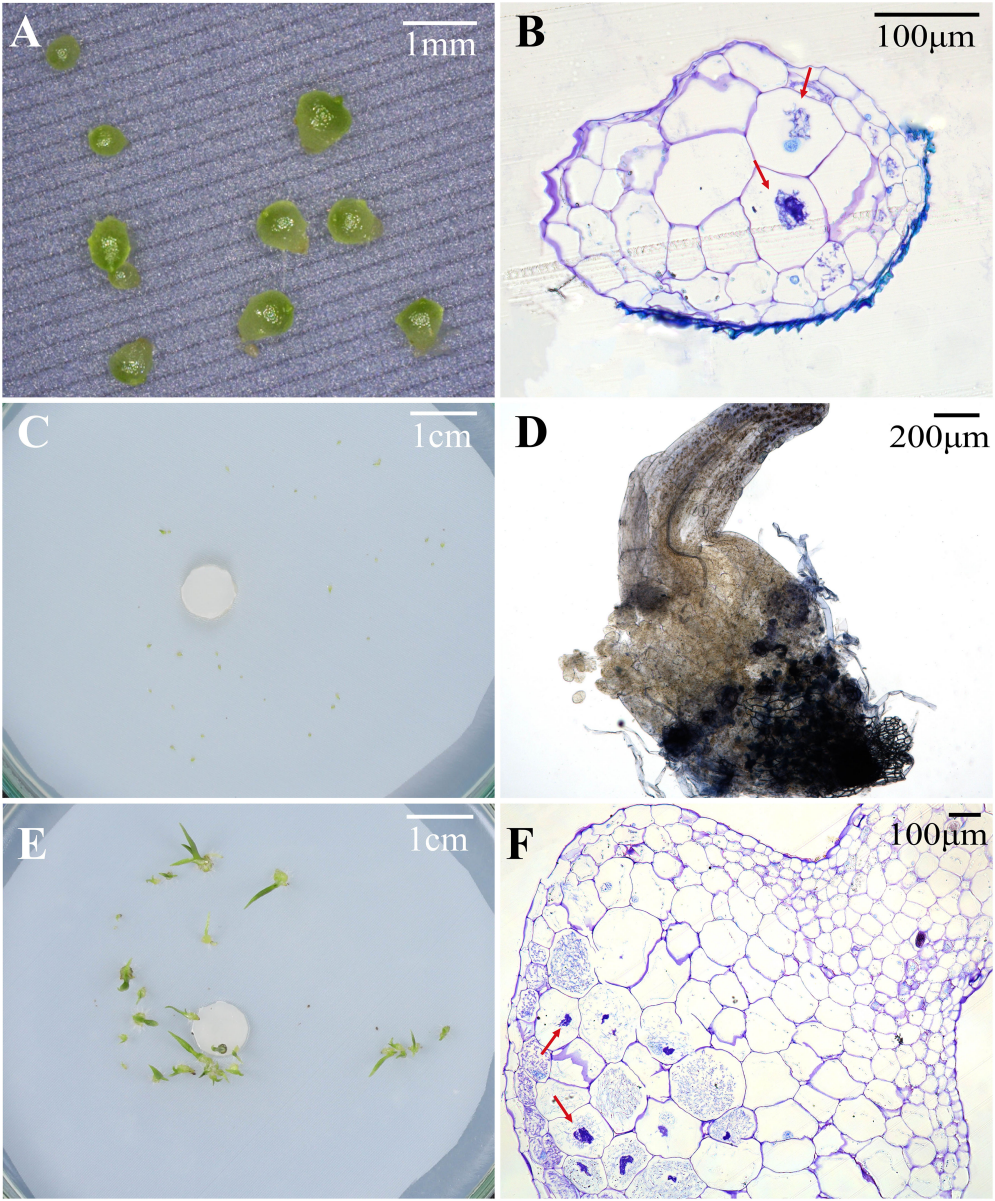
Protocorms or seedlings, status of fungal hyphae colonization, and cross-sections of protocorm or seedling showing pelotons under different fungal treatments at 30, 60 and 90 days after incubation in *Pleione bulbocodioides*. **(A)** Protocorms in SO, SI, MS or OMA treatments at 30 days after incubation; **(B)** The red arrows indicate pelotons inside the cells of a protocorm at 30 days after incubation SI treatment. **(C)** At 60 days after incubation, some already germinated seeds were no longer continue to differentiate and gradually die in MS and SI treatments; **(D)** The fungal hyphae clustered on seedling surfaces and grew into seedlings in SI treatment at 60 days after incubation; **(E)** Seedlings with two leaves in SO and SI treatments at 90 days after incubation; **(F)** A cross-sections of seedlings showing pelotons inside basal cells of seedlings in SO and SI treatments at 90 days after incubation.

At 30 days after incubation, seeds of *P. bulbocodioides* germinated in all six treatments, and the percentage of seed germination in MS treatment (91.38 ± 2.0%) was significantly higher than in other five treatments (all *P*<0.001; **Figure 2A**). At this stage, a few protocorms had already formed in SO, SI, MS and OMA treatments (**Figures 2B**, **3A**), but no seedlings were found in all treatments (**Figure 2C**). The percentage of protocorms in fungal SI treatment (9.58 ± 2.3%) was significantly higher than in fungal SO treatment (3.45 ± 3.4%; *P*<0.001), and pelotons were also observed in the basal cells of the protocorm in SI treatment (**Figure 3B**).

At 60 days after incubation, seed germination was increased in GYBQ01, Agp-1, SO and OMA treatments, while no further germination was observed in MS and SI treatments (**Figure 2D**). Some of the germinated seeds on MS and SI treatments gradually become necrotic (**Figure 3C**). At this stage, protocorms (Stages 2 or 3) formed in all treatments. The highest protocorm formation occurred in MS treatment (51.72 ± 6.0%), following by SI treatment which yielded 34.48 ± 5.3% of protocorms which was significantly higher than the other treatments (all *P* < 0.05; **Figure 2E**). A few seedlings were found in SO (0.34 ± 0.8%), SI (1.72 ± 1.0%) and MS (0.69 ± 0.5%) treatments (**Figure 2F**), and massive fungal hyphae were observed clustered on seedling surfaces and grew into seedlings in SI treatment (**Figure 3D**).

At 90 days after incubation, seed germination was decreased in all treatments while in SI treatment seed germination was recorded 37.93 ± 3.4% (**Figure 2G**). The percentage of seed germination in SI was very close to that of the percentage of protocorm formation (35.17 ± 3.8%), indicating that almost all the seeds germinated earlier developed in to protocorms or seedlings. At this stage, the percentages of protocorm formation were significantly higher in the two control treatments than in the four fungal treatments (all *P* < 0.05; **Figure 2H**). Meanwhile, seedlings were developed in SO, SI and MS treatments (**Figure 2I**). The percentage of seedlings in SO treatment (34.83 ± 3.4%) was significantly higher than in SI treatment (27.59 ± 3.5%) and MS treatment (18.39 ± 2.0%), respectively (all *P* < 0.05; **Figure 2I**). Most seedlings in SO and SI treatments bore two leaves (Stage 5; **Figure 3E**) and pelotons inside basal cells of seedlings (Stage 5; **Figure 3F**), while seedlings in MS treatment only developed one leaf.

### Asymbiotic and symbiotic seed germination of *Bletilla striata*

**Figure 4:**
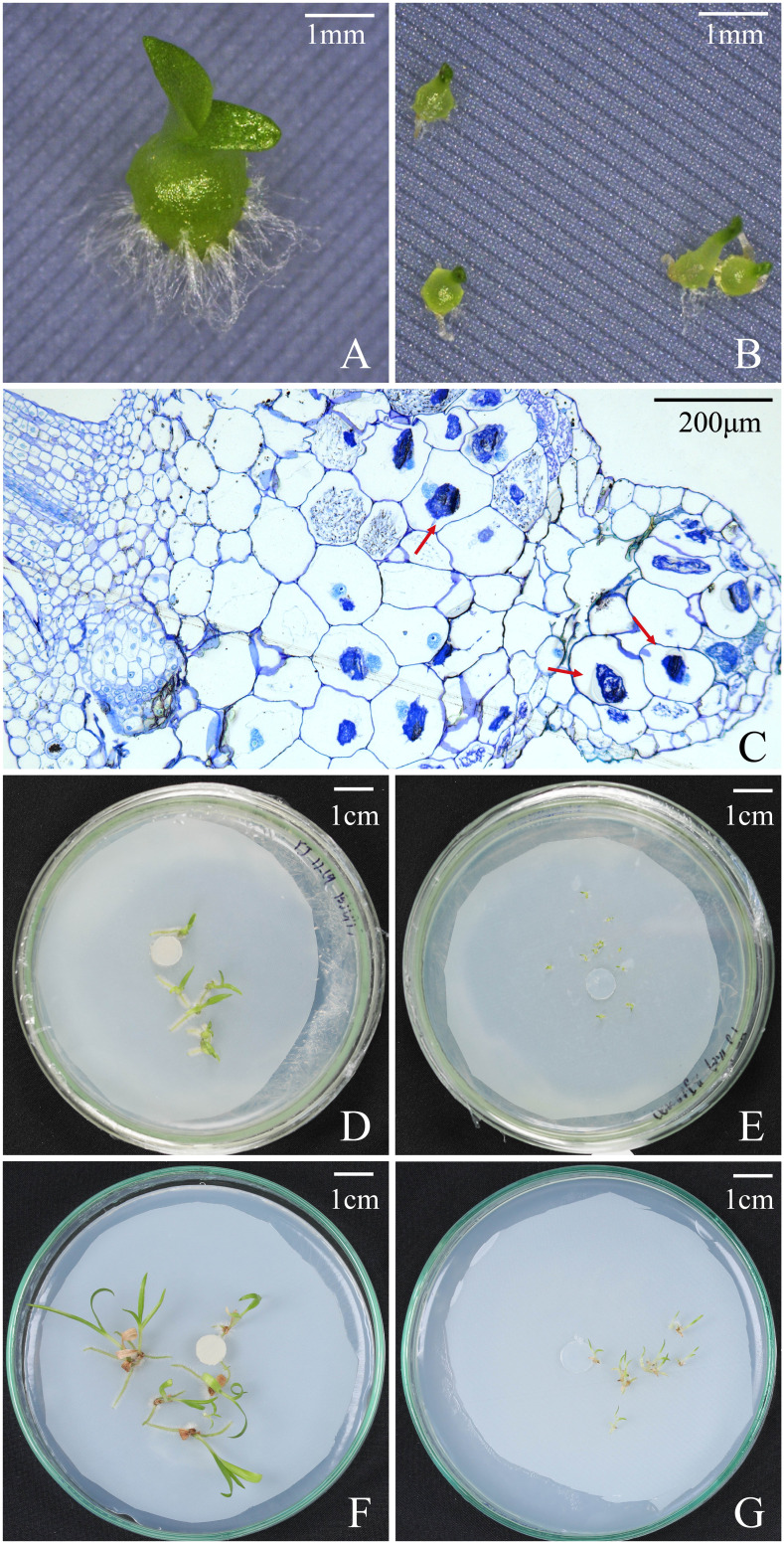
Protocorms, seedlings, and cross-sections of seedling showing pelotons under fungal SI treatment and OMA treatment at different time points after incubation in *Bletilla striata.* **(A)** Seedling in SI treatment at 30 days after incubation; **(B)** Protocorms at 30 days after incubation OMA treatment. **(C)** A cross-sections of seedlings showing pelotons inside basal cells of seedling in SI treatment at 30 days after incubation; **(D)** Seedlings in SI treatment at 60 days after incubation; **(E)** Seedlings in OMA treatment at 60 days after incubation; **(F)** Seedlings in SI treatment at 90 days after incubation; **(G)** Seedlings in OMA treatment at 90 days after incubation.

Overall, seeds of *B. striata* could germinate up to seedlings on OMA media with or without fungus SI, but seedlings developed much quickly in fungal SI treatment. At 30 days after incubation, the percentages of seed germination, protocorm formation and seedling development in fungal SI treatment were all significantly higher than in OMA treatment, respectively (**Table 2**). In fungal SI treatment, 72.92 ± 3.9% seeds already developed into seedlings (Stage 4; **Figure 4A**), and at the same time point, most of seeds in OMA treatment were still in protocorm stage (Stage 3; **Figure 4B**). Many pelotons have been observed inside the basal cells of seedlings in fungal SI treatment (**Figure 4C**).

**Table 2:** The percentages of seed germination, protocorm formation and seedling development (mean ± SE) in *Serendipita indica* (SI) and nutrient-poor medium OMA treatments of *Bletilla striata* at 30, 60 and 90 days after incubation.

Treatments	30 d			60 d			90 d		
	Seed germination	Protocorm formation	Seedling development	Seed germination	Protocorm formation	Seedling development	Seed germination	Protocorm formation	Seedling development
OMA	76.92 ± 3.7^*^	71.53 ± 4.3^**^	10.42 ± 4.2^***^	84.37 ± 2.9	84.17 ± 2.6	72.92 ± 3.9^*^	87.50 ± 4.8^***^	87.50 ± 4.8^***^	85.18 ± 3.7^**^
SI	81.94 ± 3.4^*^	77.91 ± 4.3^**^	72.92 ± 3.9^***^	82.01 ± 3.1	82.01 ± 3.1	78.70 ± 4.4^*^	77.90 ± 4.1^***^	77.90 ± 4.1^***^	77.90 ± 4.1^**^

The significant differences between two treatments were analyzed based on one-way ANOVA and indicated by ^***^ p < 0.001; ^**^ p < 0.01; ^*^ p < 0.05.

At 60 days after incubation, there were no significant differences on the percentages of seed germination and protocorm formation between the two treatments, but the percentage of seedling development in fungal SI treatment was significantly higher than on OMA treatment (*P* < 0.05; **Table 2**). At this stage, seedlings showed great differences in quality between the two treatments. All seedlings in fungal SI treatment had already developed two or three leaves with strong roots (beyond Stage 5; **Figure 4D**), while most of seedlings in OMA treatment were much smaller and just bore one or two leaves (Stage 4; **Figure 4E**).

At 90 days after incubation, all germinated seeds in SI treatment had developed into seedlings as indicated by the same percentages of seed germination, protocorm formation and seedling development, and SI treatment finally yielded 77.90 ± 4.1% seeds germinated into seedlings (**Table 2**). At the same time, the percentage of seedlings in OMA treatment reached 85.18 ± 3.7%, which was significantly higher than in SI treatment (*P* < 0.01; **Table 2**). However, the seedlings in SI treatment were obviously larger than seedlings from OMA treatment (**Figure 4F, G**).

## Discussion

Orchids normally produce an enormous quantity of dust-like seeds, the tiny seeds only have a small and undeveloped embryo without an endosperm, and highly rely on specific mycorrhizal fungi to provide mineral and carbon resources for germination and subsequent seedling growth (Arditti, 1967; Dearnaley, 2007; Rasmussen and Rasmussen, 2009). Orchids recruit mycorrhizal fungi from the so-called rhizoctonia aggregate, a polyphyletic group of fungi belonging to Tulasnellaceae, Ceratobasidiaceae and Serendipitaceae (Dearnaley et al., 2012; Weiß et al., 2016). Hence, optimal source of fungal mycobionts is essential for conservation practices through symbiotic germination (Yang et al., 2020; Wang et al., 2021).

### Effectiveness of different fungi on seed germination and seedling development in *Pleione bulbocodioides*

In this study, four fungi were used to test their abilities to promote seed germination in *P. bulbocodioides*. Overall, in fungal GYBQ01 and Agp-1 treatments, seeds only reached protocorm stage (Stage 3) until 90 days after incubation, while in fungal SO and SI treatments, seeds germinated and quickly developed into seedlings (Stage 4 and 5). The two control treatments well matched to the two situations occurred in fungal treatments, in nutrient-poor OMA treatment, seeds can germinate and form protocorms but no seedling differentiation; while in nutrient-rich MS treatment, seedlings differentiated quickly (**Figure 2**).

In a previous study, 9 fungal strains, obtained from roots of *P. bulbocodioides* and strains belong to *Trichoderma*, *Paecilomyces* and *Fusarium*, were used to assess their effects on *P. bulbocodioides* seed germination (Yang et al., 2008). All 9 fungal strains could stimulate seeds germinated till protocorm formation (Stage 2 or Stage 3), but no seedlings were observed in all treatments (Yang et al., 2008). In orchid symbiotic germination, compatible fungi could effectively promote germination up to seedlings, while incompatible fungi may stimulate germination but do not support subsequent seedling development (e.g., Zi et al., 2014; Rasmussen et al., 2015; Ma et al., 2022). The hyphae of compatible fungi could quickly colonize seeds and form pelotons to continuously provide nutrients supporting seedling development (Ma et al., 2022). In current study, pelotons were observed inside the basal cells of seedlings in SO and SI treatments (**Figures 3D, F**). The results showed that the fungi SO and SI were compatible to *P. bulbocodioides*, and seeds of *P. bulbocodioides* completely relayed on compatible fungi to germinate and develop into seedlings.

The two compatible fungal strains SO and SI had different effects on seedlings development and growth. At 60 days after incubation, many germinated seeds were found no longer continue to differentiate and gradually die in SI treatments, as well as in MS treatment, which resulted in a reduction of the total percentages of seed germination (**Figure 2**). The possible reason could be the seeds germinated quickly in the two treatments leaving limited nutrients in a petri dish failed to continuously support all germinated seeds developing into seedlings. This still need to be confirmed in future studies. Finally, the highest seedling ratio occurred in SO treatment, and hence the strain SO was the most effective symbiont to promote seed germination and seedling development in *P. bulbocodioides*.

All four fungi strains used in this study were not isolated originally from *P. bulbocodioides*. Two *Tulasnella* fungi, GYBQ01 and Agp-1, were originally isolated from two terrestrial orchids *Paphiopedilum spicerianum* and *Arundina graminifolia*, and were incompatible to *P. bulbocodioides*; while two compatible fungi, *Serendipita officinale* (SO) was originally isolated from epiphytic orchid *Dendrobium officinale* and *Serendipita indica* (SI) was obtained from rhizosphere soil (**Table 1**). Although, many studies suggested that the most efficient fungal isolates for seed germination were not necessarily those isolated originally from these orchids (e.g., Fracchia et al., 2014; Fuji et al., 2020; Zhang et al., 2020). Even the strain SO could promote 34.83 ± 3.4% seeds germinated into seedling in 90 days after incubation, its performance under the nature conditions in its original habitat is yet to be studied.

Under nature conditions, orchid mycorrhizal fungi (OMFs) live as saprobes in soil around the roots or on tree bark around epiphytic orchids (Weiß et al., 2016; Selosse et al., 2021), and their occurrence is bounded by specific habitat conditions that is related to ecological specificity (Porras-Alfaro and Bayman, 2007; Herrera et al., 2019). Orchids may associate with a wide range of mycorrhizal fungi, and the broadening and/or changing of a mycorrhizal association may enable orchids to adapt to the varied physiological changes during seed germination and seedling development (Těšitelová et al., 2012). The patchy distribution of mycorrhizal fungi can affect key processes such as seed germination, plant growth and survival, and host-fungal compatibility may be influenced by environmental factors (Harley and Smith, 1983; Jacquemyn et al., 2012). For the conservation purpose, orchid plants should be grown with mycorrhizal fungi tailored to the recipient site (Reiter et al., 2018; Wang et al., 2021). With increasing studies, using *in situ/ex situ* seed baiting to capture fungi has been considered efficient and easy way to obtain ecological/habitat-specific fungi for seed germination, in which the fungi obtained from naturally formed protocorms or seedlings tend to effectively promote seeds germination up to seedlings (e.g., Rasmussen and Whigham, 1993; Brundrett et al., 2003; Zi et al., 2014; Meng et al., 2019; Wang et al., 2021). This is also need to be done for *P. bulbocodioides*.

### Asymbiotic and symbiotic seed germination of *Bletilla striata*

It is commonly known that all orchids require mycorrhizal fungi for germination, but exceptions always occur in orchids. The results of current study clearly showed that seeds of *B. striata* could spontaneously germinate up to seedlings on the nutrient-poor OMA medium without any fungi. Seeds of *B. striata* were significantly larger than seeds of *P. bulbocodioides* (**Figure 1**), and this may also result from a large amount of starches and lipids reserved in the seeds of *B. striata* (Guo and Xu, 1990).

However, in fungal SI treatment, seeds of *B. striata* germinated and developed much quickly with 72.92 ± 3.9% seeds already developed into seedlings at 30 days after incubation, while only 10.42 ± 4.17% seedlings occurred in OMA treatment (**Table 2**). Finally, at 90 days after incubation, the percentage of seedlings in OMA treatment was significantly higher than the SI treatment (**Table 2**), however the seedlings were much weak in OMA treatment (**Figure 4G**). Because the two treatments were all conducted on OMA medium and nutrients in OMA medium can be used by fungi but not by plants, seeds of *B. striata* only depended their own nutrient reserves to germinate and develop into seedlings in OMA treatment, while in SI treatment, seeds could continuously obtain exogenous nutrients via symbiosis with fungus. The pelotons inside basal cells of seedlings were observed at 30 days after incubation (**Figure 4C**), indicated that the symbiosis had already established at this time.

The fungus *Serendipita indica* was not originally isolated from orchids (Verma et al., 1998), but fungi of *Serendipita* is considered typical OMFs (Dearnaley et al., 2012). *S. indica* has been well-studied as a root-colonizing fungus that confers diverse beneficial effects on a broad range of host plants (Zuccaro et al., 2011; Qiang et al., 2012), and our recent study also revealed that *S. indica* associated with different orchids such as *Dendrobium* and *Cymbidium*, and well promote seed germination up to seedling development (Xu et al., 2023).

Many previous studies reported that a wide range of fungi including orchid mycorrhizal fungi (OMF) and non-OMFs could promote seed germination and seedling development in *B. striata* (e.g., Yamamoto et al., 2017; Xu et al., 2019; Fuji et al., 2020; Xi et al., 2020; Liu et al., 2022). However, we don’t know if all these fungi could establish symbiosis with seeds of *B. striata*, and if not, how did fungi transfer nutrients to seeds and support seed germination and seedling development? Some of these studies may ignore that seeds of *B. striata* contain nutrients and can spontaneously germinate up to seedlings. The results of current study supported that *B. striata* seeds easily germinated without any fungi, but compatible fungi can speed seed germination and promote seedling development (Guo and Xu, 1992). The symbiotic mechanisms between orchids and fungi are still unclear but may involve fungal effector and plant receptor genes similar in plant-pathogen interactions (Favre-Godal et al., 2020). During symbiotic seed germination of *Dendrobium catenatum* with *Serendipita indica*, hypoxia-responsive genes, such as those encoding alcohol dehydrogenase (ADH), are highly induced in symbiotic protocorms, suggesting that ADH and its related hypoxia-responsive pathway are involved in establishing successful symbiotic relationships in germinating orchids (Xu et al., 2023). In the recent study on another medicinal orchid *Gymnadenia conopsea*, it revealed that bio-active steroids may play a crucial role in the symbiotic germination (Shi et al., 2023).

Nowadays, seeds sowing directly to produce seedlings has also been successfully applied in practices (Zhang et al., 2019). According to our investigation, this low-cost method has replaced *in vitro* seed germination to produce massive seedling and sped the development of Bai-Ji industry in Yunnan Province of China. Under the natural conditions, *B. striata* seeds may associate with different soil fungi to obtain exogenous nutrients, and we could use some compatible fungi, for example *S. indica*, in the practices of seeds sowing directly to speed seedling developments and improve seedling qualities.

## Conclusions

Seeds of *B. striata* do not need “helps” from any mycorrhizal fungi for germination and seedling development, but compatible fungus *S. indica* (SI) can greatly speed germination and promote seedling development. Unlike *B. striata*, seeds of *P. bulbocodioides* completely relayed on compatible fungi to germinate up to seedlings. Both fungi SO and SI were compatible to *P. bulbocodioides*, but strain SO showed stronger abilities on promoting seed germination and supporting seedling development than strain SI. Obtaining compatible fungi and using fungi to facilitate seed germination in practice are two key steps for orchid population recovery or imitation of wild cultivation based on symbiotic seed germination. Recently, a new method of seed-fungus complexes, in which orchid seeds and specific fungi are embedded together to form granules used as propagules, has been developed and considered useful for conservation of terrestrial or lithophytic orchids (Yang et al., 2023). Based on the results of current study, we suggested that fungi *Serendipita officinale* and *S. indica* could be used to produce seed-fungus complexes for the conservation or imitation of wild cultivation in *P. bulbocodioides* and *B. striata*, respectively.

## Data availability statement

The original contributions presented in the study are included in the article/supplementary material. Further inquiries can be directed to the corresponding author.

## Author contributions

JY: Writing – original draft, Methodology, Investigation, Formal analysis, Data curation. N-QL: Writing – original draft, Methodology, Investigation, Data curation. J-YG: Writing – review & editing, Writing – original draft, Supervision, Resources, Project administration, Funding acquisition, Formal analysis, Conceptualization.
